# Preparation of Lutein-Loaded PVA/Sodium Alginate Nanofibers and Investigation of Its Release Behavior

**DOI:** 10.3390/pharmaceutics11090449

**Published:** 2019-09-02

**Authors:** Xinxu Han, Peipei Huo, Zhongfeng Ding, Parveen Kumar, Bo Liu

**Affiliations:** 1 School of Materials Science and Engineering, Shandong University of Technology, Zibo 255000, China; 2 Laboratory of Functional Molecules and Materials, School of Physics and Optoelectronic Engineering, Shandong University of Technology, Xincun West Road 266, Zibo 255000, China; 3 College of Life Sciences, Shandong University of Technology, Zibo 255000, China

**Keywords:** lutein, nanofibers, polyvinyl alcohol, sodium alginate

## Abstract

This investigation aims to study the characteristics and release properties of lutein-loaded polyvinyl alcohol/sodium alginate (PVA/SA) nanofibers prepared by electrospinning. In order to increase PVA/SA nanofibers’ water-resistant ability for potential biomedical applications, the electrospun PVA/SA nanofibers were cross-linked with a mixture of glutaraldehyde and saturated boric acid solution at room temperature. The nanofibers were characterized using scanning electron microscopy (SEM) and X-ray diffractometer (XRD). Disintegration time and contact angle measurements testified the hydrophilicity change of the nanofibers before and after cross-linking. The lutein release from the nanofibers after cross-linking was measured by an ultraviolet absorption spectrophotometer, which showed sustained release up to 48 h and followed anomalous (non-Fickian) release mechanism as indicated by diffusion exponent value obtained from the Korsmeyer–Peppas equation. The results indicated that the prepared lutein-loaded PVA/SA nanofibers have great potential as a controlled release system.

## 1. Introduction

Lutein, also known as “plant lutein”, is a natural pigment and an excellent antioxidant [1] widely found in vegetables, flowers, fruits, and certain algae organisms. The intake of a certain amount of lutein as a food supplement can prevent a series of organ aging-related diseases [2]. Lutein is susceptible to light, heat, and pH [3], the property of which compromises its bioavailability and limits its storage and human administration [4]. The delivery of drugs via nano-carriers is a highly effective and proven method to improve the bioavailability and until now, numerous nano-carriers including nanofibers, nanocapsules, liposomes, polymer micelles, and nanogels have been widely investigated for the delivery of various drugs.

Electrospinning has attracted more and more attention from the past decade due to its potential use in biomedical materials, filtration, catalysis, optoelectronics, food engineering, cosmetics, and drug delivery devices [5,6,7]. Among which drug delivery is one of the most promising applications. Nanofibers produced by electrospinning exhibit several interesting properties, including high surface area to volume ratio, and void fraction [8], which make electrospinning nanofibers an appropriate candidate as a drug delivery system. Polymeric matrices such as polyvinyl alcohol (PVA), fibrinogen, chitosan, polycaprolactone, and polyvinylpyrrolidone provide an excellent source for electrospinning based on their biocompatibility [9,10]. PVA nanofibers have been widely utilized as potential biomaterials owing to its extraordinary hydrophilicity, biocompatibility and mechanical properties [11,12,13,14]. This type of material readily composes into the film due to the fact that it contains a large amount of –OH groups, which provide a platform for hydrogen bond formation with water molecules. Based on the excellent properties of PVA, much interest in research has been devoted to its electrospinning for utilization in different areas such as a biosensor [15], antimicrobial fibers [16,17,18], composite films [19,20], nanoporous films, and filtration membranes [21,22,23]. Sodium alginate (SA) is a non-toxic, biodegradable, compatible, and sustained-release material. SA shows a good hemostatic effect in combination with calcium ions, therefore, is widely used in hemostatic dressings and wound permeate absorption dressings [24]. However, the fact that SA alone in aqueous solution is not readily electrospun into a nanofiber mat and its brittleness largely restricts its application. Blending PVA and SA is an effective polymer solution that can be electrospun into nanofiber mats and in addition, their mechanical property and thermal stability can be improved probably owing to hydrogen bond formation [25]. Various studies have been reported on the development of wound dressing with PVA and SA [26,27,28,29,30]. The nanofibers made from blending PVA and SA are highly hydrophilic.

Electrospun PVA/SA nanofibers as drug carriers are limited due to the burst release of drugs. For example, Li et al. [31] reported the preparation of a fast-dissolving drug delivery system using PVA as a polymeric carrier, in which the drug was released from the nanofiber matrix in an explosive manner. In order to realize sustained release as required for some drug delivery system, it is necessary to adjust the hydrophilicity of the nanofiber. Cross-linking is one of the methods that allow drugs to be released in a controlled manner by adjusting the hydrophilicity of nanomaterials [32,33,34]. For example, Zhang et al. [35] cross-linked electrospun gelatin nanofibers with glutaraldehyde saturated steam at room temperature to improve nanofibers thermal and mechanical properties. Kenawy et al. [36] studied the controlled release of ketoprofen from electrospun PVA nanofibers with methanol cross-linking. Zhang et al. [37] used salicylic acid-loaded collagen (COL)/PVA electrospun nanofibers cross-linked with UV-radiation or glutaraldehyde to control the release of salicylic acid.

In this study, a controlled drug delivery system was developed from electrospun PVA/SA nanofibers. Lutein was utilized as a model drug. Lutein-loaded nanofibers were cross-linked by using glutaraldehyde (GA) and saturated boric acid solution as a cross-linking agent. The properties of nanofibers before and after cross-linking and the lutein release behavior were investigated.

## 2. Experimental Methods

### 2.1. Materials

Polyvinyl alcohol (PVA, 9002-89-5(CAS No.), 87%–89%(purity), Mw = 72600–81400), sodium alginate (SA, 9005-38-3), glutaraldehyde (GA, 111-30-8, 50% aqueous solution), boric acid (10043-35-3, 99.8%), sodium phosphate dibasic (Na_2_HPO_4_, 10039-32-4, 99%), potassium phosphatemonobasic (KH_2_PO_4,_ 7778-77-0, 99%), potassium chloride (KCl, 7447-40-7, 99.98%), sodium chloride (NaCl, 7647-14-5, 99.9%), and *N*,*N*-dimethylformamide (DMF, 4472-41-7, 99.5%) were purchased from Aladdin, Shanghai China. Lutein (127-40-2, 82.35%) was provided by Shandong Tian Yin Biotechnology co.ltd, Zibo, China.

### 2.2. Preparation of Nanofibers

#### 2.2.1. Preparation of Polymer Solutions

In a typical preparation, 1.6 g of PVA and 0.1 g of SA were dissolved in 15 mL of deionized water at 60 °C under constant stirring for 6 h, then cooled to room temperature. Lutein (51 mg, 3% weight ratio PVA and SA) was dissolved in 5 mL DMF at room temperature. The blended above PVA/SA solution and lutein solution were mixed and stirred for 5 h at room temperature to ensure homogeneous distribution.

#### 2.2.2. Electrospinning Lutein-Loaded PVA/SA Nanofibers

Electrospinning was performed to fabricate lutein-loaded PVA/SA nanofibers at room temperature. The mixed solution was poured into a 10 mL plastic syringe with a needle having an inner diameter of 0.41 mm. Output voltage applied to the solution was 15 kV. Besides, the flow rate of the injection pump was set to 0.3 mL/h. The nanofibers collector was cylindrical and covered by aluminum foil. At the same time, the distance from the syringe needle to the receiver collector was 150 mm. After completing the electrospinning process, the nanofibers were placed in a vacuum oven for 12 h to remove residual traces of solvents.

#### 2.2.3. Cross-Linking of Lutein-Loaded PVA/SA Nanofibers

Cross-linking of the electrospun lutein-loaded nanofibers were carried out using a mixture of GA and saturated boric acid solution. Electrospun lutein-loaded nanofibers were carefully peeled from the aluminum foil and weighed exactly using a digital balance. A weight of 0.020 g of each sample was immersed in the cross-linking fluid at room temperature for various times (1 h, 3 h, and 5 h) to carry out cross-linking. After completing the required cross-linking time for each sample, all the samples were dried with filter paper and then exposed to a vacuum oven for 12 h at room temperature to remove residual GA and water.

### 2.3. Characterization

#### 2.3.1. Morphology

The morphology of the lutein-loaded PVA/SA nanofibers before and after cross-linking was observed by quanta 250 field emission environment scanning electron microscope (SEM). The average diameter of the lutein-loaded PVA/SA nanofibers before cross-linking was calculated based on the SEM image. The distribution of lutein was observed by fluorescence microscope (CKX41, Olympus, Tokyo, Japan).

#### 2.3.2. Water Contact Angle Analysis

The surface static contact angles of the lutein-loaded PVA/SA nanofibers before and after cross-linking were investigated using a contact angle meter analysis system (JY-82, Dingsheng, Chengde, China).

#### 2.3.3. X-Ray Diffractometer Analysis

X-ray diffractometer (XRD, Bruker AXS, Karlsruhe, Germany) was used to observe the physical state of lutein in PVA/SA nanofibers in the range from 5° to 50°.

#### 2.3.4. FTIR Spectroscopy

The cross-linking effectiveness of lutein-loaded PVA/SA nanofiber mat was analyzed using Fourier transform infrared (FTIR) spectroscopy (Nicolet5700, Waltham, MA, USA).

### 2.4. Pharmacotechnical Properties

#### 2.4.1. Determination of Drug Encapsulation Efficiency

To determine the encapsulation efficiency (EE) of lutein in PVA/SA nanofibers, the lutein-loaded PVA/SA nanofibers were completely dissolved in water and lutein content was measured using UV-Vis spectrophotometer. The encapsulation efficiency of lutein was determined using the following equation:

EE (%) = real lutein content in nanofibers/theoretical lutein content in nanofiber × 100.(1)

#### 2.4.2. In Vitro Drug Release

The release profile of lutein from the cross-linked lutein-loaded PVA/SA nanofibers was studied in phosphate buffer saline (PBS, pH = 7.4) solution. Lutein-loaded PVA/SA nanofibers (20 mg) were placed in 50 mL of PBS solution at 37 °C with constant stirring. At defined time intervals, 1 mL of sample was taken from the release medium and replaced with fresh PBS to maintain the original volume. The amount of lutein released at different time intervals in PBS solution was measured by a UV-Vis spectrophotometer (UV-3600plus, Shimadzu, Kyoto, Japan). The lutein release percentage was calculated and release profile was drawn. All the measurements were performed in triplicates.

## 3. Results and Discussion

### 3.1. Morphology Characterization and XRD Spectroscopy of PVA/SA Nanofibers

**Figure 1:**
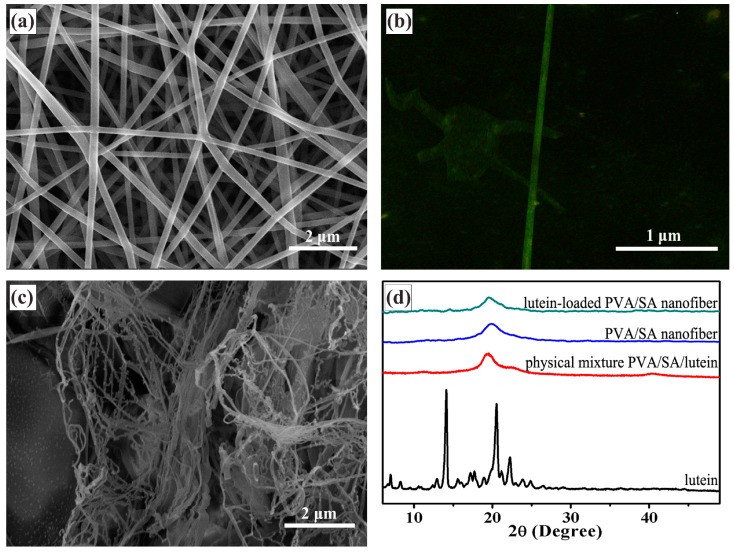
(**a**) SEM and (**b**) fluorescence microscope image of lutein-loaded polyvinyl alcohol/sodium alginate (PVA/SA) nanofibers before cross-linking, (**c**) SEM of lutein-loaded PVA/SA nanofibers after cross-linking for 1 h, (**d**) XRD diffraction pattern of free lutein, PVA/SA nanofibers, physical mixture of lutein and PVA/SA, and lutein-loaded PVA/SA nanofibers.

SEM images of un-crosslinked lutein-loaded PVA/SA nanofibers are shown in Figure 1a, displaying uniform one dimensional nanofibers with no beads and diameters in the range of 240 nm to 340 nm. Figure 1b shows the fluorescence micrograph of un-crosslinked lutein-loaded PVA/SA nanofibers. It can be observed that lutein was uniformly distributed along the axis of the nanofibers. Figure 1c shows the morphology of lutein-loaded PVA/SA nanofibers with 1 h cross-linking. Nanofibers collapse during the cross-linking, after which nanofibers were not smooth and appeared in an independent form. The morphology change indicates that the cross-linking agent induced adhesion between the electrospun nanofibers, which could be attributed to the nanofibers that tend to swell in the presence of a crosslinker and adhere with each other. In addition, visual observation showed that after the cross-linking treatment, the nanofiber mats became yellowish and shrank slightly in size, which could be due to the interaction of hydroxyl groups on the PVA with GA of the cross-linking agent [31,38]. It should be noted that the residual trace amount of GA after cross-linking treatment can induce toxicity due to its reaction with proteins. The toxicity can be eliminated via reaction with glycine [34,39].

X-ray diffraction (XRD) measurements were used to study the physical state and distribution of the lutein-loaded PVA/SA nanofibers. The XRD patterns of free lutein, PVA/SA nanofibers, and lutein-loaded PVA/SA nanofibers are shown in Figure 1d. Free lutein exhibited two strong crystal diffraction peaks between 10° and 25° (2*θ* = 20.54° and 14.06°), which were attributed to the crystallinity of lutein and consistent with the literature [40,41]. As shown in Figure 1d, the crystallization peaks were not observed in the XRD pattern of PVA/SA nanofibers, which might be due to the sensitivity of the measurement being too low to detect the crystalline drug. Since in a separate XRD measurement for physical mixture of lutein and PVA/SA in the same mass ratio as in the electrospun lutein-loaded PVA/SA nanofiber, as shown in Figure 1d, the characteristic peak of lutein crystalline structure did not appear as well.

### 3.2. Contact Angle Measurement

**Figure 2:**
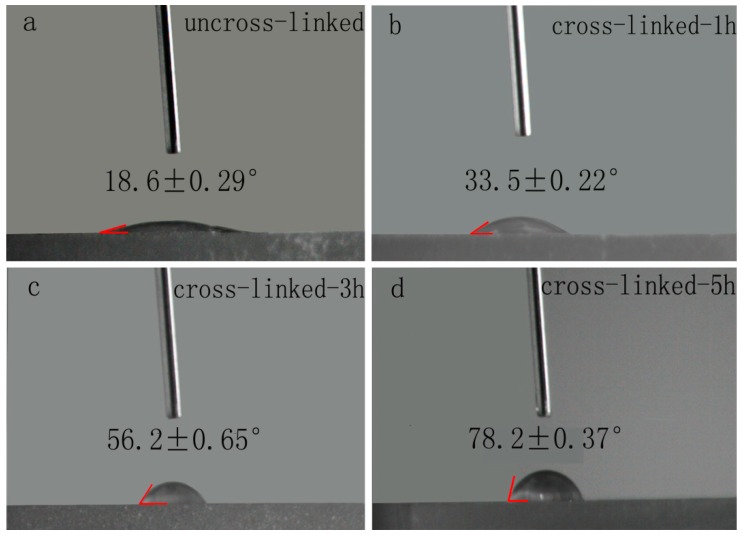
The shape of water drops and contact angle measurement for lutein-loaded PVA/SA nanofibers: (**a**) Un-crosslinked, (**b**) cross-linked for 1 h, (**c**) cross-linked for 3 h, and (**d**) cross-linked for 5 h.

Hydrophilic assessment of biological materials is a very important parameter in the field of drug delivery. Therefore, in order to determine the hydrophilicity of the nanofibers, the water contact angle of the lutein-loaded PVA/SA nanofibers before and after cross-linking was measured using a contact angle meter analysis system (Figure 2). The un-crosslinked PVA/SA nanofibers exhibited the contact angle of 18.6 ± 0.29°, which indicates that they had good hydrophilicity. Whereas the contact angle values of 33.5 ± 0.22°, 56.2 ± 0.65°, and 78.2 ± 0.37° were observed for the lutein-loaded PVA/SA nanofibers cross-linked for 1 h, 3 h, and 5 h, respectively. The cross-linked PVA/SA nanofibers showed an increase in contact angle compared to un-crosslinked PVA/SA nanofibers indicating that cross-linking could improve the hydrophilicity of nanofibers and thereby improve the stability of nanofibers in aqueous media.

### 3.3. FTIR Analysis for Cross-Linking Degree

**Figure 3:**
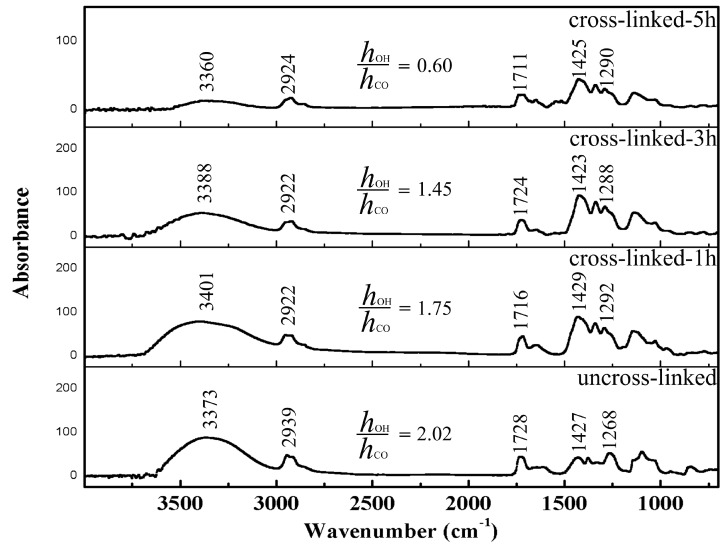
FTIR spectra for lutein-loaded PVA/SA nanofibers as un-crosslinked, cross-linked for 1 h, cross-linked for 3 h, and cross-linked for 5 h.

The cross-linking effectiveness of lutein-loaded PVA/SA was analyzed using FTIR. Infrared scanning was performed in the range of 4000 to 600 cm^−1^. The FTIR spectra of the lutein-loaded PVA/SA nanofibers with different cross-linking time are presented in Figure 3. It can be observed that spectra of lutein-loaded PVA/SA nanofibers consists prominent peaks at 3373 cm^−1^ is ascribed to a hydroxyl group, at 2939 cm^−1^ to C–H stretching (CH_2_), at 1728 cm^−1^ and 1268 cm^−1^ to acetate groups (C=O and C–O, respectively), and at 1427 cm^−1^ to C–H stretching (CH_3_) [42]. The broad peak of the hydroxyl group at 3373 cm^−1^ is due to the hydrogen bonding between hydroxyl groups of PVA and SA [43]. During the cross-linking of lutein-loaded PVA/SA with GA, the amount of hydroxyl functions decreases to create acetal functions, while the peak at 1728 cm^−^^1^ (C=O) remains constant [42]. Therefore, the ratio between signal intensity at 3373 and 1728 cm^−^^1^ could be an indicator of cross-linking degree [42,44]. The ratio between the maximum intensity of hydroxyl (h_OH_) and carbonyl functions (h_CO_) decreased from 2.02 to 0.60 as the nanofibers cross-linking time increased from 1 to 5 h. This reflects a higher cross-linking effectiveness in the lutein-loaded PVA/SA nanofibers.

### 3.4. Disintegration Characterization and In Vitro Drug Dissolution

**Figure 4:**
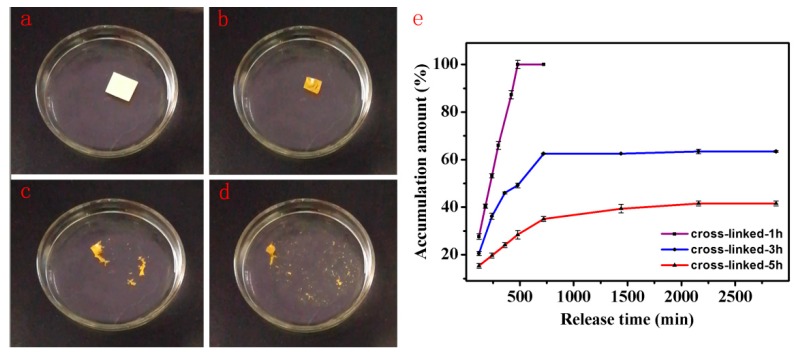
(**a**–**d**) The visual observation of the disintegration of lutein-loaded PVA/SA nanofibers, and (**e**) percentage released of lutein from electrospun PVA/SA nanofibers with different cross-linking time (1 h, 3 h, and 5 h).

The lutein-loaded PVA/SA nanofibers were cut into a size of 1 cm × 1 cm and dissolved in deionized water to verify its disintegration time. As shown in Figure 4a–d, when the lutein-loaded PVA/SA nanofibers were placed in deionized water, they first floated on the surface. Then the color of the lutein-loaded nanofibers became darker and the size contracted within 1 s, as the water molecules rapidly penetrated into the PVA/SA nanofibers scaffold, indicating the lutein release in a rush manner. As the nanofibers scaffold continued to immerse in deionized water, it rapidly disintegrated and dispersed into hundreds of small pieces (disintegration of about 3 s), which gradually dissolved in deionized water. The whole dissolving process was completed within about 7 min. The visual observation of immediate dissolving of lutein-loaded PVA/SA nanofibers is consistent with the strong hydrophilicity of PVA/SA composite.

The EE and in vitro drug release assessment is necessary in order to determine the bioavailability and extent of drug assimilation, which subsequently determines the drug therapeutic efficiency [45]. The EE of lutein-loaded PVA/SA nanofibers was found to be 91.9% ± 2.58%, which is comparable with ciprofloxacin-loaded PVA/SA formulation (EE = 98%) [43].

Since both PVA and SA polymers are hydrophilic, the composite of these two elements (i.e., PVA/SA nanofibers) by electrospinning is readily soluble in water, as shown by the disintegration test. The slow release of lutein can be achieved by cross-linking the PVA/SA nanofibers with a cross-linking agent. In order to study the effect of different levels of cross-linking on the release behavior of lutein from PVA/SA nanofibers, an in vitro release study was performed for 48 h in PBS (pH = 7.4; Figure 4e). Drug release from nanofibers can be attributed to three channels including drug desorption from the surface, proliferation of pores, and/or matrix degradation. All these steps are likely to get affected by the choice of polymer, porosity, morphology, and geometry of nanofibers [46]. For the lutein-loaded PVA/SA nanofibers cross-linked for 1 h, lutein was released in a controlled manner with an average release rate of 12.5%/h and complete lutein was released in 10 h. The controlled release performance achieved with lutein-loaded PVA/SA nanofibers cross-linked for 1 h was better in terms of release time span compared to complete release within 7 h in literature [43], which could be attributed to the usage of cross-linking agent. For the lutein-loaded PVA/SA nanofibers cross-linked for 3 h, the release rate was apparently decreased to around 9.4%/h, which was further decreased to 0.85%/h for the nanofibers cross-linked for 5 h. It can be seen from the above experimental results that drug release was dependent on cross-linking time i.e., the longer the cross-linking time, the better the sustained release of drug, which was consistent with the previous results by Zhang et al. [37].

### 3.5. The Release Kinetics Studies

The release curves were fitted to kinetic models to analyze the kinetics of in vitro drug release. It was proved that lutein was uniformly distributed in the PVA/SA nanofibers. Lutein was loaded into the polymer matrix by a simple packaging of the polymer, the kinetics of drug release in PVA/SA nanofibers was analyzed using the Korsmeyer–Peppas kinetic model and the Higuchi model (matrix system) respectively.

The Korsmeyer–Peppas equation is as follows:

M_*t*_/M_∞_ × 100% = *kt^n^*, (2)

where, M_*t*_ is the mass of the released drug at *t* time, M_∞_ is the mass of the released drug when the time approaches infinity, *k* is a constant, and *n* is the diffusion exponent. This expression depicts a proportional mass release out of the polymer matrix with time. The value of *n* is dependent on the type of drug delivery mechanism, geometry, and polydispersity. When *n* < 0.5, 0.5 < *n* < 1.0, and 0.5 < *n* < 1.0, the type of release follows Fickian diffusion, non-Fickian diffusion, and Case-II transport, respectively.

Higuchi model is another widely used pattern for analyzing the mechanism of drug release:

M_*t*_/M_∞_ × 100% = *k*_1_*t*^1/2^, (3)

where *k*_1_ is the diffusion rate constant.

Table 1 shows values of *n*, *k*, *k*_1_, and the correlation coefficient (*R*^2^) from fitting curves with two models. The *R*^2^ values of the Korsmeyer–Peppas model (matrix system) were closer to 1, compared to that of the Higuchi model for all the three nanofibers with different cross-linking time. From the Korsmeyer–Peppas model, *n* values were found to be 0.7398, 0.5840, and 0.6278 for lutein release from PVA/SA nanofibers cross-linked for 1 h, 3 h, and 5 h, respectively, indicating that lutein release from PVA/SA nanofibers occurred through non-Fickian (anomalous) diffusion suggesting that more than one mechanism process was involved in lutein release. These mathematical models could be purely empirical through the insignificant changes of *R*^2^. Although the release mechanism requires further clarification, the phenomenon of the study proves that drugs can be released from the electrospun PVA/SA nanofibers matrices in a continuous manner.

The release of the drug in the drug-loaded nanofibers is controlled by the diffusion of drug within the polymer matrix and/or matrix degradation, which involves bulk and surface-polymer erosion, depending on the polymer composition [47]. When the penetration of water into the nanofiber matrix is slower than the matrix degradation, surface erosion predominates and when matrix degradation is faster than water penetration, bulk erosion predominates. In addition, water penetration into the individual nanofibers can affect the drug release as well. In our case, the initial drug release from the hydrophilic PVA/SA nanofibers cross-linked for 1 h, was primarily determined by the diffusion of drug and dissolution of the polymer due to the penetration of water. However, nanofibers become more hydrophobic when cross-linked for 5 h, which was verified by water contact angle measurements. Due to the enhanced hydrophobicity of the PVA/SA nanofibers, the degradation of the polymer matrix occurred relatively slow, at this point the release of the drug in the nanofibers was primarily determined by the diffusion of drug.

## 4. Conclusions

In this research, the lutein-loaded PVA/SA nanofibers with uniform and smooth morphology were obtained by electrospinning. The PVA/SA nanofibers were cross-linked for different time points and their hydrophilicity was measured with a contact angle measurement experiment. XRD analysis showed that lutein was present in the stable amorphous state in the PVA/SA nanofibers. The sustained release was achieved after the tuning the hydrophilicity of the lutein-loaded PVA/SA nanofibers as it released the lutein in a controlled manner extending up to 48 h. The drug release kinetics revealed that the release of the lutein was through non-Fickian diffusion mechanism. The results indicate that encapsulation of lutein utilizing polymer matrices by electrospinning is an effective method in drug delivery and cross-linking could further help to achieve the sustained lutein release by tuning the hydrophilicity. Therefore, the drug-loaded PVA/SA nanofibers developed in this study has great potential to be used as the delivery system in the near future.

## Figures and Tables

**Table 1:** The release of lutein from PVA/SA nanofibers cross-linked for 1 h, 3 h, and 5 h.

PVA/SA Nanofibers Cross-Linking Time	Korsmeyer–Peppas Model		Higuchi Model	
1 h	*k* *n* *R* ^2^	24.97 ± 2.713 0.7398 ± 0.0653 0.9707	*k*_1_ *R* ^2^	36.19 ± 1.736 0.8993
3 h	*k* *n* *R* ^2^	23.08 ± 2.056 0.5840 ± 0.0608 0.9641	*k*_1_ *R* ^2^	25.94 ± 0.7468 0.9560
5 h	*k* *n* *R* ^2^	12.81 ± 1.358 0.6278 ± 0.0709 0.9540	*k*_1_ *R* ^2^	15.32 ± 0.5497 0.9334

## References

[1] Silva J.T.P., Geiss J.M.T., Oliveira S.M., Brum E.S., Sagae S.C., Becker D., Leimann F.V., Ineu R.P., Guerra G.P., Gonçalves O.H. (2017). Nanoencapsulation of lutein and its effect on mice’s declarative memory. Mater. Sci. Eng. C.

[2] Sato Y., Kobayashi M., Itagaki S., Hirano T., Noda T., Mizuno S., Sugawara M., Iseki K. (2011). Protective effect of lutein after ischemia-reperfusion in the small intestine. Food Chem..

[3] Wang Y.F., Ye H., Zhou C.H., Lv F.X., Bie X.M., Lu Z.X. (2012). Study on the spray-drying encapsulation of lutein in the porous starch and gelatin mixture. Eur. Food Res. Technol..

[4] Steiner B.M., Mcclements D.J., Davidov-Pardo G. (2018). Encapsulation systems for lutein: A review. Trends Food Sci. Technol..

[5] Bhardwaj N., Kundu S.C. (2011). Silk fibroin protein and chitosan polyelectrolyte complex porous scaffolds for tissue engineering applications. Carbohydr. Polym..

[6] Agarwal S., Greiner A., Wendorff J.H. (2013). Functional materials by electrospinning of polymers. Prog. Polym. Sci..

[7] Nagarajan S., Pochat-Bohatier C., Balme S., Miele P., Kalkura S.N., Bechelany M. (2017). Electrospun fibers in regenerative tissue engineering and drug delievery. Pure Appl. Chem..

[8] Opanasopit P., Sila-On W., Rojanarata T., Ngawhirunpat T. (2013). Fabrication and properties of capsicum extract-loaded PVA and CA nanofiber patches. Pharm. Dev. Technol..

[9] Yang J.M., Yang J.H., Tsou S.C., Ding C.H., Hsu C.C., Yang K.C., Yang C.C., Chen K.S., Chen S.W., Wang J.S. (2016). Cell proliferation on PVA/sodium alginate and PVA/poly (γ-glutamic acid) electrospun fiber. Mater. Sci. Eng. C.

[10] Jia Y.T., Wu C., Dong F.C., Huang G., Zeng X.H. (2012). Preparation of PCL/PVP/Ag Nanofiber Membranes by Electrospinning Method. Appl. Mech. Mater..

[11] Khatri Z., Wei K., Kim B.-S., Kim I.-S. (2012). Effect of deacetylation on wicking behavior of co-electrospun cellulose acetate/polyvinyl alcohol nanofibers blend. Carbohydr. Polym..

[12] Vashisth P., Nikhil K., Roy P., Pruthi P.A., Singh R.P., Pruthi V. (2016). A novel gellan-PVA nanofibrous scaffold for skin tissue regeneration: Fabrication and characterization. Carbohydr. Polym..

[13] Fathi-Azarbayjani A., Qun L., Chan Y.W., Chan S.Y. (2010). Novel vitamin and gold-loaded nanofiber facial mask for topical delivery. AAPS PharmSciTech.

[14] Deng Y., Zhang X., Zhao Y., Liang S., Xu A., Gao X., Deng F., Fang J., Wei S. (2014). Peptide-decorated polyvinyl alcohol/hyaluronan nanofibers for human induced pluripotent stem cell culture. Carbohydr. Polym..

[15] Ren G., Xu X., Liu Q., Cheng J., Yuan X., Wu L., Wan Y. (2006). Electrospun poly (vinyl alcohol)/glucose oxidase biocomposite membranes for biosensor applications. React. Funct. Polym..

[16] Ignatova M., Starbova K., Markova N., Manolova N., Rashkov I. (2006). Electrospun nano-fibre mats with antibacterial properties from quaternised chitosan and poly (vinyl alcohol). Carbohydr. Res..

[17] Wang Y., Yang Q., Shan G., Wang C., Du J., Wang S., Li Y., Chen X., Jing X., Wei Y. (2005). Preparation of silver nanoparticles dispersed in polyacrylonitrile nanofiber film spun by electrospinning. Mater. Lett..

[18] Lee H.K., Jeong E.H., Baek C.K., Youk J.H. (2005). One-step preparation of ultrafine poly (acrylonitrile) fibers containing silver nanoparticles. Mater. Lett..

[19] Duan B., Yuan X., Zhu Y., Zhang Y., Li X., Zhang Y., Yao K. (2006). A nanofibrous composite membrane of PLGA-chitosan/PVA prepared by electrospinning. Eur. Polym. J..

[20] Shao C., Kim H.-Y., Gong J., Ding B., Lee D.-R., Park S.-P. (2003). Fiber mats of poly (vinyl alcohol)/silica composite via electrospinning. Mater. Lett..

[21] Hong Y., Shang T., Li Y., Wang L., Wang C., Chen X., Jing X. (2006). Synthesis using electrospinning and stabilization of single layer macroporous films and fibrous networks of poly (vinyl alcohol). J. Membr. Sci..

[22] Li L., Hsieh Y.-L. (2006). Chitosan bicomponent nanofibers and nanoporous fibers. Carbohydr. Res..

[23] Qin X.-H., Wang S.-Y. (2006). Filtration properties of electrospinning nanofibers. J. Donghua Univ..

[24] Pasparakis G., Bouropoulos N. (2006). Swelling studies and in vitro release of verapamil from calcium alginate and calcium alginate–chitosan beads. Int. J. Pharm..

[25] Ni P.L., Bi H.Y., Zhao G., Han Y.C., Wickramaratne M.N., Dai H.L., Wang X.Y. (2019). Electrospun preparation and biological properties in vitro of polyvinyl alcohol/sodium alginate/nano-hydroxyapatite composite fiber membrane. Colloids Surf. B.

[26] Kamoun E.A., Kenawy E.R.S., Tamer T.M., El-Meligy M.A., Eldin M.S.M. (2015). Poly (vinyl alcohol)-alginate physically crosslinked hydrogel membranes for wound dressing applications: Characterization and bio-evaluation. Arab. J. Chem..

[27] Ishikawa K., Ueyama Y., Mano T., Koyama T., Suzuki K., Matsumura T. (1999). Self-setting barrier membrane for guided tissue regeneration method: Initial evaluation of alginate membrane made with sodium alginate and calcium chloride aqueous solutions. J. Biomed. Mater. Res..

[28] Niranjan R., Kaushik M., Selvi R.T., Prakash J., Venkataprasanna K.S., Prema D., Pannerselvam B., Venkatasubbu G.D. (2019). PVA/SA/TiO_2_-CUR patch for enhanced wound healing application: In vitro and in vivo analysis. Int. J. Biol. Macromol..

[29] Kim J.O., Choi J.Y., Park J.K., Kim J.H., Jin S.G., Chang S.W., Li D.X., Hwang M.-R., Woo J.S., Kim J.-A. (2008). Development of clindamycin-loaded wound dressing with polyvinyl alcohol and sodium alginate. Biol. Pharm. Bull..

[30] Kim J.O., Park J.K., Kim J.H., Jin S.G., Yong C.S., Li D.X., Choi J.Y., Woo J.S., Yoo B.K., Lyoo W.S. (2008). Development of polyvinyl alcohol–sodium alginate gel-matrix-based wound dressing system containing nitrofurazone. Int. J. Pharm..

[31] Li X., Kanjwal M.A., Lin L., Chronakis I.S. (2013). Electrospun polyvinyl-alcohol nanofibers as oral fast-dissolving delivery system of caffeine and riboflavin. Colloid Surf. B.

[32] Hu X., Liu S., Zhou G., Huang Y., Xie Z., Jing X. (2014). Electrospinning of polymeric nanofibers for drug delivery applications. J. Control. Release.

[33] Nagarajan S., Soussan L., Bechelany M., Teyssier C., Cavaillès V., Pochat-Bohatier C., Miele P., Kalkura N., Janot J., Balme S. (2016). Novel biocompatible electrospun gelatin fiber mats with antibiotic drug delievery properties. J. Mater. Chem. B.

[34] Nagarajan S., Belaid H., Pochat-Bohatier C., Teyssier C., Iatsunskyi I., Coy E., Balme S., Cornu D., Miele P., Kalkura N.S. (2017). Design of boron nitride/gelatin electrospun nanofibers for bone tissue engineering. ACS Appl. Mater. Interfaces.

[35] Zhang Y.Z., Venugopal J., Huang Z.M., Lim C.T., Ramakrishna S. (2006). Crosslinking of the electrospun gelatin nanofibers. Polymer.

[36] Kenawy E.R., Abdel-Hay F.I., El-Newehy M.H., Wnek G.E. (2007). Controlled release of ketoprofen from electrospun poly (vinyl alcohol) nanofibers. Mater. Sci. Eng. A.

[37] Zhang X., Tang K., Zheng X. (2016). Electrospinning and crosslinking of COL/PVA nanofiber-microsphere containing salicylic acid for drug delivery. J. Biogic. Eng..

[38] Vashisth P., Pruthi V. (2016). Synthesis and characterization of crosslinked gellan/PVA nanofibers for tissue engineering application. Mater. Sci. Eng. C.

[39] Li X.C., Yan S.S., Dai J., Lu Y., Wang Y.Q., Sun M., Gong J.K., Yao Y. (2018). Human lung epithelial cells A549 epithelial-mesenchymal transition induced by PVA/collagen nanofiber. Colloids Surf. B.

[40] Hadipour-Goudarzi E., Montazer M., Latifi M., Aghaji A.A.G. (2014). Electrospinning of chitosan/sericin/PVA nanofibers incorporated with in situ synthesis of nano silver. Carbohydr. Res..

[41] Muhoza B., Zhang Y., Xia S., Cai J., Zhang X., Su J. (2018). Improved stability and controlled release of lutein-loaded micelles based on glycosylated casein via Maillard reaction. J. Funct. Foods.

[42] Gonzalez-Ortiz D., Pochat-Bphatier C., Gassara S., Camdedouzou J., Bechelany M., Miele P. (2018). Development of novel h-BNNS/PVA porous membranes via Pickering emulsion templating. Green Chem..

[43] Kataria K., Gupta A., Rath G., Mathur R.B., Dhakate S.R. (2014). In vivo wound healing performance of drug loaded electrospun composite nanofibers transdermal patch. Int. J. Pharm..

[44] M’barki O., Hanafia A., Bouyer D., Faur C., Sescousse R., Delabre U., Blot C., Guenoun P., Deratani A., Quemener D. (2014). Greener method to prepare porous polymer membranes by combining thermally induced phase separation and crosslinking of poly (vinyl alcohol) in water. J. Membr. Sci..

[45] Vashisth P., Raghuwanshi N., Srivastava A.K., Singh H., Nagar H., Pruthi V. (2017). Ofloxacin loaded gellan/PVA nanofibers-Synthesis, characterization and evaluation of their gastroretentive/mucoadhesive drug delivery potential. Mater. Sci. Eng. C.

[46] Thakkar S., Misra M. (2017). Electrospun polymeric nanofibers: New horizons in drug delivery. J. Pharm. Sci..

[47] Kajdič S., Planinšek O., Gašperlin M., Kocbek P. (2019). Electrospun nanofibers for customized drug-delivery systems. J. Drug Deliv. Sci. Technol..

